# Translation, psychometric evaluation and validation of the “diabetes health profile-18” questionnaire in Arabic

**DOI:** 10.11604/pamj.2021.40.212.31410

**Published:** 2021-12-08

**Authors:** Maali Haoues, Chekib Zedini, Molka Chadli-Chaieb

**Affiliations:** 1 Faculty of Medicine Ibn El Jazzar of Sousse, University of Sousse, Sousse, Tunisia,; 2 Department of Community Medicine, Faculty of Medicine Ibn El Jazzar of Sousse, University of Sousse, Sousse, Tunisia,; 3 Department of Endocrinology and Metabolic Diseases, Farhat Hached University Hospital, Sousse, Tunisia

**Keywords:** Diabetes, quality of life, translation, validation, psychometric properties, diabetes health profile-18

## Abstract

**Introduction:**

measuring quality of life requires an instrument validated in the population language. The purpose of our study was to translate and analyze the psychometric properties of the literary Arabic version of the “diabetes health profile (DHP)-18”.

**Methods:**

we conducted a methodological study for psychometric evaluation and validation of the DHP-18, following the steps of the cross-cultural validation described by Vallerand. A convenience sample of people with diabetes was collected for this purpose. The developed questionnaire included participants‘ demographic characteristics, diabetes data and the experimental version of the DHP-18 questionnaire. Validity, reliability and questionnaire standards establishment were carried out.

**Results:**

a sample of 333 diabetics was recruited. Test-retest correlation coefficient (r = 0.985; p<0.01) and Cronbach's alpha coefficient (alpha = 0.840) showed that the experimental version was accurate in terms of temporal stability and internal consistency. The content validity index was 0.84 and showed that the questionnaire statements accurately measured the concepts under study. The exploratory principal axis factoring, using the orthogonal varimax rotation, allowed the extraction of a factorial solution with four independent factors, grouping the 18 items of the questionnaire. Correlation coefficients between the three corresponding dimensions of the theoretical model of the questionnaire were low and positive, between 0.431 and 0.535, confirming that each dimension measured a unique content.

**Conclusion:**

the literary Arabic version of the DHP-18 has proven to be valid, reliable and ready for use in clinical practice in Tunisian people with diabetes.

## Introduction

Diabetes mellitus is a serious chronic disease that affects many aspects of people´ life [1]. Its worldwide prevalence is significantly increasing according to the World Health Organization and the International Diabetes Federation estimations [2,3]. A 96% increase in the number of people with diabetes between 2019 and 2045 is expected in the Middle East and North Africa Region [3]. Tunisia is at risk to reach an annual increase of 11.7% in 2030 [4]. Acute and chronic complications of diabetes can potentially affect people´s lives, namely its social and family life. Diabetes mellitus complications can lead to functional handicap, work discontinuation [5] and expose to premature death [6,7]. They increase health system care costs [2,3]. As people´ quality of life depends on health care´s quality, professionals have to strive to improve people with diabetes management [8]. Measuring quality of life requires an instrument validated in the population language. The majority of available instruments are designed and validated in English for American and British populations [9,10]. Few of them have been validated in Arabic. The purpose of our study was to translate and analyze the psychometric properties of the literary Arabic version of the “diabetes health profile-18” (DHP-18), which is read and understood by all Arabic-speaking populations, in order to use it in Tunisian people with diabetes.

## Methods

**Research design:** we conducted a methodological study of DHP-18´s psychometric evaluation and validation. Our approach was based on the cross-cultural validation technique described by Vallerand [11].

**Study setting:** data were collected from November 1^st^, 2019 to March 15^th^, 2020, in public health establishments and basic health centers that agreed to participate in the study, in Sousse (Tunisia): the Endocrinology and Metabolic Disease, Internal Medicine, Cardiology and Dermatology Departments of Sahloul and Farhat Hached University Hospitals of Sousse and in eight basic health centers of Sousse to reach a more heterogeneous population.

**Participants:** we opted for convenience sampling based on the following inclusion criteria: type 1 or type 2 participants with diabetes, whose disease lasts one year or more, aged 18 years and over and able to read and understand a newspaper in Arabic. Any participant with cognitive impairment or altered mental status, detected by the “Mini-Health State Examination (MMSE)” in its Tunisian version [12], was excluded from the study.

**Data collection:** the developed questionnaire contained two sections. The first one was intended to collect participants‘demographic characteristics (age, sex, civil status, socioeconomic status and habitat) as well as diabetes data (type, duration, complications and treatment). The second section contained the DHP-18 itself, which is a multidimensional and self-administered questionnaire that assesses the psychological and behavioral functioning of people with diabetes through three dimensions: psychological distress (6 items), barriers to activity (7 items) and disinhibited eating (5 items) [13]. Instrument´s rating is carried out according to an algorithmic formula that takes into account the three dimensions of the questionnaire [14].

**Translation and validation process:** in our study, the DHP-18 translation and validation process lasted 16 months, from March 8^th^, 2019, to July 1^st^, 2020. The method took place in seven steps: 1/preparation of preliminary versions by two parallel reversed translations (2 translations from English to Arabic and 2 from Arabic to English); 2/evaluation of the preliminary versions and preparation of an experimental version by a first panel of 13 experts in endocrinology, translation and languages; 3/carrying out a pre-test of this version with 30 Tunisian diabetics and making changes and reformulations, after the approval of the committee members; 4/validation of the content of the questionnaire according to the modified Delphi method [15], by a second panel of 6 experts: 4 endocrinologists and 2 epidemiologists. The clarity and relevance of the questions´ statements are judged by calculating the content validity index (CVI); 5/administration of the questionnaire to diabetics for the evaluation of internal consistency, with the Cronbach´s alpha coefficient. The questionnaire was also administered twice, 1 month apart (test-retest), to the same group of diabetics, for the study of temporal stability and the measurement of reproducibility; 6/evaluation of the validity of the construct on the responses provided by diabetics, by means of a principal analysis factoring (PAF) via orthogonal varimax rotation and the study of the relationships between the three corresponding dimensions of the theoretical model of the DHP-18. If one of validity steps did not meet the Vallerand standards [11], the items not satisfying the statistics were reformulated and then retested on a new sample of people with diabetes, before being validated again by the second panel of experts after CVI´s questionnaire recalculation; 7/the process was ended by the establishment of questionnaire standards tables [11].

**Statistical analysis:** the statistical study was conducted using SPSS software version 21.0. Categorical variables were expressed as relative frequency (%). The quantitative ones were summarized by measurements of central trend (mean: M) and dispersion (standard deviation: SD) when following normal law or by median and interquartile range (25^th^ quartile and 75^th^ quartile). The Content Validity Index (CVI) was calculated by dividing the number of items with a score of 3 and 4 by the total number of items. A CVI of 0.80 or greater indicates acceptable validity [16]. The study of internal consistency was explored using the Cronbach´s alpha formula, of which a value of 0.5 is acceptable and values ranging from 0.70 to 0.85 are desirable [9]. Pearson´s r-correlation coefficient between test and retest scores at 1-month intervals was interpreted as satisfactory according to Vallerand [11] if positive and ≥ 0.60. The intra-class correlation coefficients (ICC) was calculated between test and retest response scores, with a 95% confidence interval, and interpreted as follows: very good if ICC ≥ 0.91; good if 0.90 ≤ ICC ≤ 0.71; moderate if 0.70 ≤ ICC ≤ 0.51; low if 0.50 ≤ ICC ≤ 0.31 and very low if ICC ≤ 0.30 [17]. The significance level was set at p ≤ 5%. In order to measure the responsiveness, Cohen's d coefficient was tested by comparing the mean difference in the response scores between test and retest, divided by their standard deviation. Values of d are considered to have a “small” effect, indicating satisfactory reproducibility, for a value of 0.2, a “medium” effect for a value of 0.5 and a “large” effect for a value of 0.8 [18]. A significant Bartlett sphericity test (p <5%) and a Kayser-Mayer-Olkin index (KMO) greater than 0.5 were used to verify the adequacy of the correlation matrix for further exploratory PAF [11]. The validity of the construct was explored by an unforced PAF with orthogonal rotation (varimax). The relationship between the three corresponding dimensions of the theoretical model was carried out using the Pearson correlation coefficient. Positive and low correlation coefficients demonstrate that each dimension measures a single content [11]. The establishment of standards tables required the determination of percentile rank, means and standard deviations of questionnaire scores and its 3 dimensions ‘scores, and the calculation of Z and T scores so that they can be used as standards for the new translated and validated version of the DHP-18 [11].

**Ethical considerations:** the project was approved by the Human Research Ethics Committee of the Faculty of Medicine of Sousse, on July 27^th^, 2020, under the reference CEFMS 54/ 2020. A license has been obtained from the University of Oxford for the translation and validation of the original version of the DHP-18. Authorizations from the directors and head of departments of the study sites were also obtained. A consent form written in Arabic and validated by the Ethics Committee was read and signed by all persons included in the study. The copyright for the validated Arabic version of the instrument is reserved to the University of Oxford and will be provided upon request.

## Results

**Participants ‘demographic characteristics:** three hundred and thirty-three people with diabetes were included. Their average age was 51.11 (16.21) years with extremes of 18 to 90 years. The sex ratio was 0.94. Sixty-one per cent of participants had a medium socioeconomic status, 70.3% lived in urban areas and 65.5% were married.

**Diabetes data:** seventy-three percent of people with diabetes had type 2 diabetes (n = 243) and 27% had type 1. The median duration of the disease was 7 years with extremes of 1 to 38 years. Diabetes complications were present in 38.7% of the persons with diabetes and 60.7% of them were treated with insulin. Ischemic heart disease, diabetic retinopathy and diabetic foot lesions were observed in 7.2%, 6.6% and 6% of people with diabetes, respectively. All three conditions were associated in 16.2% of them.

**Reliability of the experimental version of the DHP-18:** the sample of 333 people with diabetes type 1 and type 2, selected by a convenience sample, was invited to respond twice, one month apart, to the experimental version of the DHP-18. One hundred and sixty participants responded to the questionnaire the second time, for a response rate of 48%. The test-retest correlation coefficients for the overall score and scores of the three dimensions of the experimental version of the DHP-18 showed a satisfactory temporal stability at one month (Table 1). The means and standard deviations of the dimension scores and the overall score were similar between the test and the retest (Table 1). The correlation coefficients were above the minimum threshold of 0.60. Cohen´s d coefficient between the scores of the 3 dimensions and the overall score of the questionnaire found a “small” associated effect (Table 1). The ICC values were close to 1, indicating similarity of responses within the same group in the time interval (Table 1). The internal consistency, evaluated by Cronbach´s alpha, was 0.840.

**Table 1:** test-retest of the experimental DHP-18 version (n = 160)

Dimensions /overall score	Score in test	Score in retest	Correlation coefficient	Cohen's d	ICC	95% CI
	M(SD)	M(SD)				
Psychological distress	54.17(19.08)	54.48(18.55)	0.969*	-0.098	0.984	(0.978-0.988)
Barriers to activity	58.45(14.86)	59.17(14.86)	0.984*	-0.047	0.992	(0.989-0.994)
Disinhibited eating	50.58(12.96)	50.88(12.28)	0.971*	-0.242	0.985	(0.979-0.989)
DHP -18 overall score	29.61(6.70)	29.86(6.91)	0.985*	-0.090	0.993	(0.990-0.995)

ICC: intra-class correlations coefficient; CI: confidence interval; *p<0.01

### Validity of the experimental version of the DHP-18

**Content validity:** content validity of the modified sections of the experimental version of the questionnaire was verified by a panel of six experts, who discussed the relevance and precision of the statements of all items. The instrument´s CVI was 0.84, demonstrating that the statements accurately measured the concepts explored.

### Construct validity

**Psychological dimension´s structure:** the analysis, based on 333 people with diabetes´ responses to the questionnaire, provided satisfactory results from the KMO (0.847) and Bartlett´s (p<10^-3^) tests, allowing to continue the PAF. The unforced factor analysis performed on the first 18 items of DHP-18, extracted 4 factors grouping the 18 items, which explained 62.42% of the total variance (Table 2). The items 1, 3, 8 and 14, which turned out to be complex variables on factor analysis, have been kept after confirmation of their theoretical and conceptual relevance. The scale´s Cronbach alpha was reduced after item removal (item 1 (alpha = 0.826); item 3 (alpha = 0.826); item 8 (alpha = 0.825) and item 14 (alpha = 0.824)), confirming their relevance. All items were retained for final analysis, to respect the structure of the original questionnaire and keep its homogeneity. The iterations number for the factors ‘rotation to converge was 9. The varimax orthogonal rotation resulted in 18 items divided according to 4 factors that underlie people with diabetes´ quality of life. The PAF results showed that factors 1 and 3 had the same items as the first and third dimensions of the original instrument, while a fourth factor comprising items 2 and 4 alone was extracted by the analysis. Saturation for both items was low for the second factor. The reliability of the theoretical dimensions was checked by calculating Cronbach's alpha, including items 2 and 4, in the second dimension of the questionnaire, to keep the structure of the original instrument. All Cronbach's alpha values were above the threshold of 0.5: (psychological distress; 0.852, barriers to activity; 0.670, and disinhibited eating; 0.504). Items 4 and 9 presented negative complete correlations of corrected items (-0.036 and -0.308 respectively), on analysis by the Cronbach's alpha formula and Cronbach's alpha values which increased after removing the item (0.856 and 0.863 respectively), compared to the items that preceded or succeeded. Translation of these two items was corrected after administration of the questionnaire to a new group of 10 people with diabetes and after consulting two experts in translation. Then, the expert´s panel met again to validate the content of the questionnaire. The recalculated CVI was 0.91, allowing the last version of the instrument to be determined.

**Table 2:** principal axis factoring analysis and saturation coefficients of the experimental DHP-18 version after orthogonal varimax rotation (n = 333)

	Factor 1	Factor 2	Factor 3	Factor 4
Eigen value	6.041	2.144	1.926	1.125
% explained variance	33.850	11.910	10.700	6.249
**Items**				
DHP6 (D1)	0.476			
DHP8 (D1)	0.514			
DHP15 (D1)	0.733			
DHP16 (D1)	0.854			
DHP17 (D1)	0.821			
DHP18 (D1)	0.823			
DHP5 (D3)		0.609		
DHP7 (D3)		0.504		
DHP9 (D3)		0.668		
DHP10 (D3)		0.671		
DHP12 (D3)		0.581		
DHP1 (D2)			0.771	
DHP3 (D2)			0.645	
DHP11 (D2)			-0.550	
DHP 13 (D2)			0.666	
DHP14 (D2)			0.568	
DHP2 (D2)				0.737
DHP4 (D2)				0.765

(D1), (D2), (D3): refers to the dimension of the questionnaire being explored; DHP 1 to 18 refers to items scanned

**Relationships between the three dimensions of the DHP-18´s theoretical model:** the correlation coefficients between the three dimensions of the experimental version of DHP-18 were low and positive (0.431 between disinhibited eating and psychological distress and 0.535 between barriers to activity and psychological distress).

**Establishment of DHP-18 standards:** in our study, the DHP-18 Z and T scores showed Gaussian curves: “psychological distress” (Z (-2,651; 2,372); T (23.48; 73.72)), “barriers to activity” (Z (-3,741; 2,286); T (12.59; 73.86)) and “disinhibited eating behaviors” (Z (-2,665; 2,589); T (23.35; 75.89)). The overall DHP-18 score had also a Gaussian distribution (Z (-3,584; 2,828); T (14.16; 78.28)) (Table 3). The average scores of the 3 dimensions of the questionnaire, owing to the received treatment, were higher than the standards provided by the descriptive analysis of the original version of DHP-18, conducted by Meadows [13] (Table 4).

**Table 3:** Arabic version of DHP-18, standards table (n = 333)

Percentile	Z score				T score			
	Overall score	Psychological distress	Barriers to activities	Disinhibited eating	Overall score	Psychological distress	Barriers to activities	Disinhibited eating
5	-1.589	-1.470	-1.806	-1.170	34.107	35.302	31.937	32.902
10	-1.162	-1.174	-1.355	-1.232	38.381	38.258	36.453	37.678
20	-0.763	-0.879	-0.839	-0.754	42.372	41.213	41.613	42.455
25	-0.592	-0.879	-0.516	-0.754	44.082	41.213	44.838	42.455
30	-0.449	-0.583	-0.516	-0.754	45.507	44.169	44.838	42.455
40	-0.307	-0.288	-0.194	-0.277	46.932	47.124	48.063	47.231
50	-0.218	0.008	0.129	-0.277	49.782	50.080	51.288	47.231
60	0.263	0.304	0.451	0.201	52.632	53.035	54.513	52.008
70	0.406	0.599	0.451	0.201	54.058	55.991	54.513	52.008
75	0.548	0.599	0.451	0.679	55.482	55.991	54.513	56.785
80	0.691	0.895	0.774	0.679	56.907	58.946	57.783	56.785
90	1.403	1.486	1.096	1.634	64.032	64.858	60.964	66.338
95	1.831	1.781	1.741	1.634	68.307	67.813	67.414	66.338

**Table 4:** comparative results of the 3 dimensions’ mean scores of DHP-18 questionnaire according to the received treatment between experimental and original version

Instrument	Dimension		Original version (13)	Experimental version (our study)
			M(SD)	M(SD)
DHP-18			(n=435)	(n=333)
	Psychological distress	Diet	12.9 (13.8)	53.70 (20.56)
		Tablets	21.5 (20.5)	53.36 (20.05)
		Insulin	31.0 (23.0)	53.30 (17.66)
	Barriers to activities	Diet	30.0 (22.0)	59.13 (15.66)
		Tablets	18.6 (16.1)	56.66 (16.43)
		Insulin	13.8 (12.8)	60.47 (11.99)
	Disinhibited eating	Diet	37.4 (23.4)	53.89 (15.69)
		Tablets	33.4 (23.3)	50.42 (13.35)
		Insulin	33.2 (24.8)	49.98 (13.92)

Scores of DHP-18's original version are derived from the meadows descriptive analysis

## Discussion

This survey was conducted on 333 people with diabetes type 1 and type 2 to translate and analyze the psychometric properties of the literary Arabic version of DHP-18. Results showed that the psychometric validation according to the cross-cultural validation of Vallerand [11] produced a reliable and valid Arabic version. Cronbach´s alpha coefficient was 0.840. Test-retest correlation coefficient was equal to 0.985 with p<0. 01. Cohen´s d effect of the 3 dimensions of the questionnaire was “small” (maximal value was equal to 0.242) and ICC values were close to 1. Content validity index was 0.84. The exploratory principal axis factoring, using the orthogonal varimax rotation, allowed the extraction of a factorial solution with four independent factors, grouping the 18 items of the questionnaire. Calculation of the correlation coefficients between the three corresponding dimensions of the theoretical model of DHP-18 showed that these were low and positive, between 0.431 and 0.535.

Tan *et al*. [19], in a study which aimed to analyze the psychometric properties of the original version of DHP-18 in a multiethnic population with type 2 diabetes in Singapore, found a Cronbach's alpha exceeding 0.70 for the three dimensions of the questionnaire. The study of Jelsness-Jorgensen *et al*. [20], whose aim was to translate the DHP-18 from English to Norwegian, found Cronbach's alpha greater than 0.7. In our study, a Cronbach's alpha of 0.84 was considered satisfactory. Jelsness-Jorgensen *et al*. [20] tested the responsiveness of the translated version of the questionnaire in people with diabetes whose disease state was unchanged between the test and the retest, by the ICC. For those with deterioration or an improvement in DHP-18 scores, responsiveness was calculated by the paired t test and the size of Cohen's d-effect. Quality of life was unchanged between testing and retesting in 79.2% of study participants. ICC values were 0.82, 0.76, and 0.74 for the dimensions of psychological distress, barriers to activity, and disinhibited eating. Improvement (10.1% of participants) or deterioration (10.7% of participants) of quality of life was noted in some cases, but with no statistically significant differences between the test and the retest scores of the questionnaire dimensions. Cohen´s d effects were undetectable or small. In our study, the responsiveness assessed by the ICC and the Cohen´s coefficient was unchanged between test and retest in all participants. ICC values were greater than 0.9, with “small” Cohen´s d values for the three dimensions of the questionnaire, indicating a satisfactory reliability of the Arabic version of DHP-18.

As in our study, the forced 3-factor PAF, carried out in Jelsness-Jorgensen *et al*. study [20], to keep the structure of the original instrument, did not provide the expected results. The three factors in the analysis explained only 42% of the total variance. Items 4, 13 and 14 in the second dimension “barriers to activity” had a saturation coefficient below the threshold (0.40). The unforced PAF allowed the extraction of 4 factors which explained 46% of the total variance, more in line with the original structure of the instrument. Three items of the theoretical dimension of “barriers to activity” (items 11, 13 and 14) formed a fourth factor, whose saturation´s coefficients were low for the theoretical dimension [20]. Confirmatory factor analysis was performed in the Norwegian study for model adjustment [20]. In our study, the unforced PAF improved the structure of the instrument and identified four dimensions explaining 62.42% of the total variance. The revealed fourth independent factor included items 2 and 4, whose saturation coefficients were low for the second factor. To keep the structure of the original instrument, we used the calculation of Cronbach's alpha of the theoretical dimensions as well as the reformulation and retesting of the rectified items in the PAF.

In Jelsness-Jorgensen *et al*. study [20], the correlations between the dimensions of the instrument (psychological distress, barriers to activity and disinhibited eating behaviors) ranged from 0.22 to 0.37. These low and positive correlations were consistent with those observed in our study. They estimated that each dimension measures a single content, according to the standards established by Vallerand [11]. Comparison of mean and standard deviations scores of the 3 dimensions of DHP-18 according to received treatment, to those provided by the descriptive analysis of the original version [13], allowed us to argue that Tunisian people with diabetes were not statistically comparable to English ones. The establishment of the standard table of DHP-18´s Arabic version will allow estimating life´s quality of Tunisian and other speaking and reading Arabic people with diabetes, by comparison with participants who validated the Arabic version of DHP-18 in our study (Table 3).

This study is the first in Tunisia to have translated into literary Arabic and evaluated the psychometric properties of a tool for measuring the life´s quality of people with diabetes. The produced questionnaire met the criteria of the validation process. This new instrument has psychometric properties satisfactory and comparable to those of the original version. The convergent validity could not be carried out in our study because of the language barrier, as a large part of the Tunisian population does not speak English fluently. We used the validation of the construct by PAF, the calculation of Cronbach's alpha of the theoretical dimensions, as well as the reformulation and retesting of the corrected items. Confirmatory factor analysis could not be performed in our study.

## Conclusion

This study concluded that the Arabic version of the DHP-18 has satisfactory psychometric properties comparable to those of the original version. This instrument is ready for use in clinical practice and education programs.

### What is known about this topic

- Instruments measuring quality of life of people with diabetes have been mainly designed and validated in English within American and British populations;
- The development of an Arabic version of an instrument measuring diabetics quality of life is relevant and necessary for clinical and methodological purposes.

### What this study adds

- This study is the first in Tunisia to have translated into literary Arabic and evaluated the psychometric properties of a tool for measuring quality of life of people with diabetes;
- The psychometric validation of DHP-18 in Arabic, according to the cross-cultural validation of Vallerand produced a reliable and valid Arabic version;
- This instrument is ready for use in clinical practice and education programs.

## References

[1] Gebremedhin T, Workicho A, Angaw DA (2019). Health-related quality of life and its associated factors among adult patients with type II diabetes attending MizanTepi University Teaching Hospital, Southwest Ethiopia. BMJ Open Diabetes Res Care.

[2] Cho NH, Shaw JE, Karuranga S, Huang Y, Rocha Fernandes JD, Ohlrogge AW (2018). IDF diabetes atlas: global estimates of diabetes prevalence for 2017 and projections for 2045. Diabetes Res Clin Pract.

[3] Saeedi P, Petersohn I, Salpea P, Malanda B, Karuranga S, Unwin N (2019). Global and regional diabetes prevalence estimates for 2019 and projections for 2030 and 2045: results from the International Diabetes Federation Diabetes Atlas, 9^th^edition. Diabetes Res Clin Pract.

[4] Shaw JE, Sicree RA, Zimmet PZ (2010). Global estimates of the prevalence of diabetes for 2010 and 2030. Diabetes Res Clin Pract.

[5] Gastaldi G, Oracion V, Dorribo V, Pralong JA (2019). Diabetes mellitus and work. Rev Med Suisse.

[6] Rao Kondapally Seshasai S, Kaptoge S, Thompson A, Di Angelantonio E, Gao P, Sarwar N (2011). Diabetes mellitus, fasting glucose, and risk of cause-specific death. N Engl J Med.

[7] Baldo V, Lombardi S, Cocchio S, Rancan S, Buja A, Cozza S (2015). Diabetes outcomes within integrated healthcare management programs. Prim Care Diabetes.

[8] WHO Quality of Life Assessment Group (1996). Quelle qualité de vie?. Forum mondial de la Santé.

[9] Bradley C, Todd C, Gorton T, Symonds E, Martin A, Plowright R (1999). The development of an individualized questionnaire measure of perceived impact of diabetes on quality of life: the ADDQoL. Qual Life Res.

[10] Meadows K, Steen N, McColl E, Eccles M, Shiels C, Hewison J (1996). The diabetes health profile (DHP): a new instrument for assessing the psychosocial profile of insulin requiring patients--development and psychometric evaluation. Qual Life Res.

[11] Vallerand RJ (1989). Vers une méthodologie de validation trans-culturelle de questionnaires psychologiques: Implications pour la recherche en langue française. Can Psychol Can.

[12] Bellaj T, Jemaa SB, Romdhane NA, Dhiffallah M, Ali NB, Bouaziz M (2008). Version arabe du Mini Mental State Examination (A-MMSE): fidélité, validité et données normatives. Tunis Med.

[13] Meadows KA, Abrams C, Sandbaek A (2000). Adaptation of the diabetes health profile (DHP-1) for use with patients with type 2 diabetes mellitus: psychometric evaluation and cross-cultural comparison. Diabet Med.

[14] Mulhern B, Meadows K (2014). The construct validity and responsiveness of the EQ-5D, SF-6D and diabetes health profile-18 in type 2 diabetes. Health Qual Life Outcomes.

[15] Steurer J (2011). The Delphi method: an efficient procedure to generate knowledge. Skeletal Radiol.

[16] Waltz CF, Strickland O, Lenz ER (2017). Measurement in nursing and health research. New York: Springer Publishing Company.

[17] Fermanian J (2005). Validation des échelles d´évaluation en médecine physique et de réadaptation: comment apprécier correctement leurs qualités psychométriques. Ann Readapt Med Phys.

[18] Cohen J (2013). Statistical power analysis for the behavioral sciences.

[19] Tan ML, Khoo EY, Griva K, Lee YS, Amir M, Zuniga YL (2016). Diabetes health profile-18 is reliable, valid and sensitive in Singapore. Ann Acad Med Singap.

[20] Jelsness-Jørgensen L-P, Jensen Ø, Gibbs C, Bekkhus Moe R, Hofsø D, Bernklev T (2018). Psychometric testing of the Norwegian diabetes health profile (DHP-18) in patients with type 1 diabetes. BMJ Open Diabetes Res Care.

